# Inhibition of telomerase activity in malignant glioma cells correlates with their sensitivity to temozolomide

**DOI:** 10.1038/sj.bjc.6601193

**Published:** 2003-08-26

**Authors:** T Kanzawa, I M Germano, Y Kondo, H Ito, S Kyo, S Kondo

**Affiliations:** 1 Department of Neurosurgery, Mount Sinai School of Medicine, New York, NY 10029, USA; 2 Department of Neurosurgery, The University of Texas MD Anderson Cancer Center, Houston, TX 77030, USA; 3 Department of Obstetrics and Gynecology, Kanazawa University, School of Medicine, 13-1 Takaramachi, Kanazawa, Ishikawa 920-8641, Japan

**Keywords:** temozolomide, glioma, telomerase, hTERT

## Abstract

Temozolomide (TMZ, 3,4-dihydro-3-methyl-4-oxoimidazo [5,1-d]-as-tetrazine-8-carboxamide) is a new alkylating agent with promising antitumour efficacy for malignant gliomas. The resistance of tumour cells to TMZ is primarily associated with levels of the alkylguanine alkyltransferase (AGT). O^6^-benzylguanine (O^6^-BG), an inhibitor for AGT, reduced resistance to TMZ. Recently, it has been demonstrated that chemosensitivity of tumour cells is related to a decline in telomerase activity. However, it is unknown if TMZ sensitivity of malignant glioma cells correlates with telomerase. In this study, using malignant glioma cells with low levels of AGT (U373-MG and U87-MG) and high levels of AGT (T98G), we investigated the association among AGT, telomerase, and TMZ sensitivity. U373-MG and U87-MG cells were sensitive to TMZ (IC_50_ for a 2-day treatment=100 *μ*M), while T98G cells were resistant to TMZ (IC_50_ for a 2-day treatment >500 *μ*M). Treatment with TMZ (100 *μ*M) suppressed telomerase activity in U373-MG and U87-MG cells in a time-dependent manner, but not in T98G cells. The downregulation of telomerase activity in U373-MG and U87-MG cells was due to inhibition of the human telomerase reverse-transcriptase (hTERT) gene expression at the transcriptional level. This inhibitory effect was induced by interfering with transcription factor Sp1 binding sites of the hTERT core promoter. Interestingly, O^6^-BG not only sensitised T98G cells to TMZ, but also suppressed telomerase activity. These findings suggest that response of malignant glioma cells to TMZ can be monitored by reduction in telomerase activity. Therefore, quantification of telomerase activity during or after treatment with TMZ may be a useful marker to detect treatment efficacy.

Temozolomide (TMZ, 3,4-dihydro-3-methyl-4-oxoimidazo [5,1-d]-as-tetrazine-8-carboxamide) is a new oral alkylating agent that has recently demonstrated efficacy in the treatment of malignant gliomas (O'Reilly *et al*, 1993; Plowman *et al*, 1994; Friedman *et al*, 1995, 2000; Yung *et al*, 1999). The cytotoxicity of TMZ is primarily due to the formation of O^6^-methylguanine in DNA, which mispairs with thymine during the next cycle of DNA replication (Denny *et al*, 1994; D'Atri *et al*, 1995). Subsequent futile cell cycles of DNA mismatch repair result in cell death in cancer cells (D'Atri *et al*, 1998). Therefore, the sensitivity of tumour cells to TMZ is correlated with the activity of the DNA repair protein O^6^-alkylguanine alkyltransferase (AGT), which removes alkyl groups at the O^6^ position of guanine. Cells with low levels of AGT are sensitive to TMZ, while cells expressing high levels of AGT are more resistant (Dolan *et al*, 1990; Wedge *et al*, 1996). O^6^-benzylguanine (O^6^-BG), a potent inhibitor of AGT-mediated resistant, enhances the activity of TMZ in tumour cells with high levels of AGT (Dolan *et al*, 1990; Wedge *et al*, 1996).

Recently, several investigations have demonstrated that chemotherapy-induced cell killing of tumour cells is associated with a decline in telomerase activity (Faraoni *et al*, 1997; Chou *et al*, 2001; Lin *et al*, 2001). Telomerase is a ribonucleoprotein enzyme with reverse-transcriptase activity responsible for maintenance of telomeric repeats onto the 3′-end of chromosomes (Blackburn *et al*, 1989; Harley *et al*, 1994). Three components of human telomerase have been recently identified: the RNA component (hTER) (Feng *et al*, 1995), the telomerase-associated protein (TEP1) (Harrington *et al*, 1997; Nakayama *et al*, 1997), and the telomerase reverse transcriptase (hTERT) (Meyerson *et al*, 1997; Nakamura *et al*, 1997). Although both hTER and hTERT are necessary for telomerase activity, hTERT is the major determinant of telomerase activity. The vast majority of malignant tumours express telomerase activity highly, while telomerase is not detected in most normal cells (Kim *et al*, 1994; Chadeneau *et al*, 1995; Shay and Wright, 1996; Hiraga *et al*, 1998). Therefore, telomerase is not only a useful tumour marker, but also may be a good indicator of chemotherapy response. However, it is unclear whether responsiveness of malignant glioma cells to TMZ is associated with the level of telomerase activity.

In this study, using malignant glioma U373-MG and U87-MG cells with low levels of AGT and T98G cells with high levels of AGT, we assessed the link between their sensitivity to TMZ and telomerase activity. We here demonstrate that U373-MG and U87-MG cells were sensitive to TMG and their telomerase activity was suppressed by blocking binding sites of transcription factor Sp1, which is important for hTERT. In contrast, in TMZ-resistant T98G cells, a significant decrease in telomerase activity was undetectable. However, O^6^-BG not only sensitised T98G cells to TMZ, but also inhibited telomerase activity. Taken together, our findings suggest that quantification of telomerase activity during or after TMZ treatment may be a useful marker to measure the therapeutic efficacy.

## MATERIALS AND METHODS

### Tumour cell lines

Human malignant glioma U373-MG, U87-MG, and T98G cells were purchased from ATCC (American Tissue Culture Collection, Rockville, MD, USA). Cells were cultured in Dulbecco's modified Eagle's medium (DMEM, GIBCO BRL, Grand Island, NY, USA) supplemented with 10% fetal bovine serum (GIBCO BRL), 4 mM glutamine, 100 U ml^−1^ penicillin, and 100 *μ*g ml^−1^ streptomycin. U373-MG and U87-MG cells have low levels of AGT, while T98G cells have high levels of AGT (Baer *et al*, 1993; Pieper *et al*, 1996).

### Reagent

Temozolomide was kindly supplied by the Schering-Plough Research Institute (Kenilworth, NJ, USA) and was dissolved in DMSO (Sigma Chemical Co., St Louis, MO, USA). O^6^-benzylguanine was obtained from Sigma Chemical Co. and dissolved in DMSO at 50 mM stock solution.

### Cell viability assay

The cytotoxic effect of TMZ on malignant glioma cell lines was determined by using a trypan blue dye exclusion assay as described previously (Mukai *et al*, 2000). Tumour cells were seeded at 2 × 10^3^ cells per well (0.1 ml) in 96-well, flat-bottomed plates and incubated overnight at 37°C. After exposure to TMZ at concentrations ranging from 10 to 1000 *μ*M for 2–6 days, the cells were trypsinised and the number of viable cells was counted. The viability of untreated cells was regarded as 100%. To investigate the effect of O^6^-BG, T98G cells were seeded at 2 × 10^3^ cells per well (0.1 ml) in 96-well flat-bottomed plates and 24 h later treated with 0.1% DMSO (control) or 50 *μ*M O^6^-BG in serum-free DMEM for 2 h. After that, 100 *μ*M TMZ was added and the cells were cultured for an additional 3 days.

### Cell cycle analysis

For cell cycle analysis, tumour cells treated with or without TMZ (100 *μ*M) for 2 or 3 days were trypsinised, stained with propidium iodide by using the Cellular DNA Flow Cytometric Analysis Reagent Set (Boehringer Mannheim, Indianapolis, IN, USA), and analysed for DNA content by using the FACScan (Becton Dickinson, San Jose, CA, USA) as described previously (Komata *et al*, 2000). Data were analysed by Cell Quest software (Becton Dickinson). Dead cells were gated out by using pulse processing.

### Telomerase activity (TRAP) assay

The TRAP assay was performed with TRAPEZE™ Telomerase Detection Kit (Oncor Inc.,. Gaithernburg, MD, USA) according to the manufacturer's instruction with some minor modifications (Kondo *et al*, 1998a). Cells (10^6^) were washed once in PBS and homogenised in 200 *μ*l of ice-cold lysis buffer (0.5% 3-[3-cholamidopropyl-dimethylammonio]-1-propane-sulphonate (CHAPS), 10 mM Tris-HCl, pH 7.5, 1 mM ethylenebis (oxyethylene-nitrilo) tetraacetic acid (EGTA), 5 mM *β*-mercaptoethanol, 0.1 mM 4-(2-aminoethyl) benzenesulphonyl fluoride (AEBSF), 10% glycerol). After 30 min of incubation on ice, the lysates were centrifuged at 10 000 **g** for 15 min at 4°C, and the supernatant was rapidly frozen and stored at −80°C. The concentration of protein was measured using the BioRad Protein Assay (Richmond, CA, USA). Each extract (1 *μ*g protein) was assayed in a 50 *μ*l reaction mixture containing 10 *μ*l of 5 × TRAP Reaction Mix and 2 U of *Taq* DNA polymerase (Invitrogen). After a 30-min incubation at 30°C for telomerase extension, the reaction mixture was then subjected to PCR amplification in a thermal cycler for 34 cycles at 94°C for 30 s, 55°C for 30 s. Then, 20 *μ*l of PCR products was applied to a 10% polyacrylamide gel (Bio-Rad, Richmond, CA, USA) and visualised with SYBR Green (Molecular Probe, Inc., Eugene, OR, USA) according to the manufacturer's instruction. The band intensity was measured by densitometry. The extract from U373-MG cells and the CHAPS lysis buffer were used as positive and negative control, respectively.

### RT–PCR analysis for hTR or hTERT RNA expression

The expression of hTR or hTERT mRNA was analysed by semiquantitative RT–PCR amplification that we have more recently described (Komata *et al*, 2001). The correlation between band intensity and dose of cDNA templates was linear under the conditions described below. RNA of each cell line treated with or without TMZ was isolated using the RNA Isolation Kit (QIAGEN Inc., Valencia, CA, USA). RT–PCR was performed with total RNA (0.1 *μ*g) using the ThermoScript™ RT–PCR Kit (Invitrogen, Carlsbad, CA, USA). The thermal cycles were: 94°C for 1 min, 60°C for 2 min, and 70°C for 2 min for 35 cycles for GAPDH (450 bp); 94°C for 1 min, 60°C for 1 min, and 72°C for 1 min for 30 cycles for hTR (126 bp); and 94°C for 1 min, 58°C for 1 min, and 72°C for 1 min for 30 cycles for hTERT (145 bp). The primer sets used were as follows: GAPDH, 5′-CTCAGACACCATGGGGA-AGGTGA-3′ (forward) and 5′-ATGATCTTGAGGCTGTTGTCATA-3′ (reverse); hTR, 5′-TCTAACCCTAACTGAGAAGGGCGTAG-3′ (forward) and 5′-GTTTGCTCTAGAATGAACGGTGGAAG-3′ (reverse); hTERT, 5′-CGGAAGAGTGTCTGGAGCAA-3′ (forward) and 5′-GGATGAAGCGGAGTCTGGA-3′ (reverse). The amplified products were fractionated on a 2% agarose gel containing 0.5 *μ*g ml^−1^ ethidium bromide, gels were photographed with Polaroid film (Polaroid type 667), and photographs were quantitatively scanned using the NIH image software. The efficiency of cDNA synthesis from each sample was estimated by PCR with GAPDH-specific primers.

### Luciferase assay

The transcriptional activity of hTERT in malignant glioma cells after TMZ treatment was determined by the hTERT promoter-luciferase reporter plasmids as described previously (Takakura *et al*, 1999). The structures of the hTERT promoter-luciferase constructs are shown in Figure 4B. Various lengths of DNA fragments upstream of the initiating ATG codon of the hTERT promoter gene were PCR amplified and inserted into luciferase reporter vector pGL3-Basic (Promega CORP-BTC, Madison, WI, USA). For the construction of reporter plasmids containing substitution mutations in factor binding sites of c-Myc or Sp1, site-specific mutagenesis was performed by a PCR-based protocol as described previously (Kyo *et al*, 2000). Cells were plated at a density of 5 × 10^4^ cells ml^−1^ day prior to TMZ treatment. At 1 day after exposure to TMZ, the cells were transfected with the luciferase reporter plasmids (0.5 *μ*g each) using GenePorter (Gene Therapy System INC., San Diego, CA, USA) according to the manufacturer's instruction. At 48 h after transfection, cells were washed twice with PBS and lysed in the lysis buffer provided with the luciferase kit (Promega CORP-BTC). Transcriptional activity was measured using a Microtiter Plate Luminometer (Dynatech Laboratories, Inc., Chantilly, VA, USA). To ensure that the luciferase assay was within the linear range for samples and positive control, the standard curve of light units *vs* relative enzyme concentration was obtained by making serial dilutions of luciferase (QuantiLum® Recombinant Luciferase, Promega CORP-BTC) in 1 × lysis buffer with 1 mg ml^−1^ BSA. The *β*-galactosidase reporter pCMV*β* (Promega CORP-BTC) was cotransfected as control for transfection efficiency. *β*-Galactosidase activity was measured simultaneously using the *β*-galactosidase assay kit (Promega CORP-BTC).

### Statistical analysis

Cell viability, telomerase activity measured by TRAPEZE, and relative luciferase activity represent the average of three independent assays. Statistical differences were assessed using an unpaired Student's *t*-test comparing each data point with untreated cells. A probability (*P*) <0.05 was considered significant.

## RESULT

### Sensitivity of malignant glioma cells to TMZ

**Figure 1:**
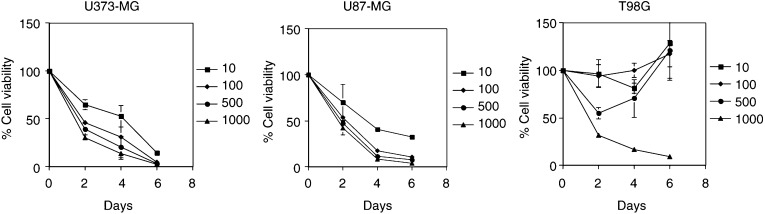
Sensitivity of malignant glioma cells to TMZ. Tumour cells were seeded at 2 × 10^3^ cells per well (0.1 ml) in 96-well flat-bottomed plates and incubated overnight at 37°C. After exposure to TMZ at concentrations ranging from 10 to 1000 *μ*M for 2–6 days, the cells were trypsinised and the number of viable cells was counted. The viability of the untreated cells was regarded as 100%. The results shown are the means±s.d. of three independent experiments.

To determine the effect of TMZ over time and increasing concentrations, tumour cells were treated with TMZ (10–1000 *μ*M) for 2–6 days. As shown in Figure 1

, treatment with TMZ inhibited the viability of U373-MG and U87-MG cells having low levels of AGT in a time- and dose-dependent manner. The CD_50_ (concentration resulting in cell viability of 50% of control) of TMZ (a 2-day treatment) for U373-MG or U87-MG cells was approximately 100 *μ*M. On the other hand, T98G cells, which have high levels of AGT, were resistant to TMZ (Figure 1). TMZ at a concentration of 10 or 100 *μ*M did not induce significant antitumour effect. The inhibitory effect of TMZ (500 *μ*M) was temporary and T98G cells started proliferating 4 days after exposure to TMZ. Treatment with TMZ at a concentration of 1000 *μ*M inhibited the viability of T98G cells in a time-dependent manner. The TMZ CD_50_ (a 2-day treatment) for T98G cells was more than 500 *μ*M, indicating that the resistance factor is more than five-fold. Therefore, we chose the concentration of 100 *μ*M to treat U373-MG, U87-MG, and T98G cells for further experiments.

### Effect of TMZ on cell cycle of malignant glioma cells

**Figure 2:**
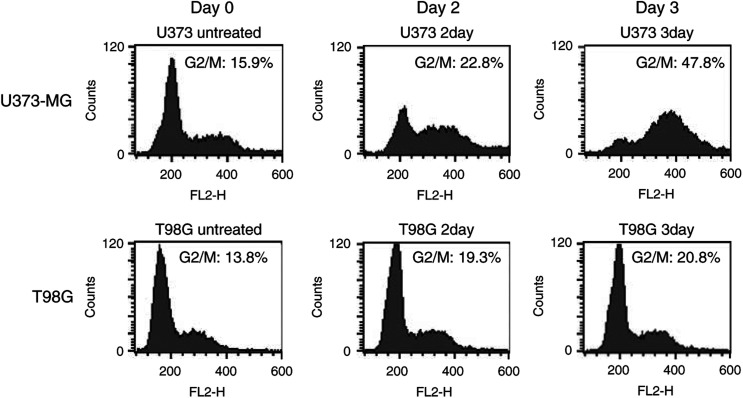
Effect of TMZ on cell cycle of U373-MG or T98G malignant glioma cells. For cell cycle analysis, tumour cells were collected 2 or 3 days after exposure to TMZ (100 *μ*M), and stained with propium iodide and analysed in the FACScan. The percentage of cells in different phases was determined by using Cell Quest software. The data shown are representative of three independent experiments.

To determine if TMZ affects cell cycle of malignant glioma cells, the DNA flow cytometric analysis was performed. As shown in Figure 2

, TMZ treatment (100 *μ*M) increased the cell population in the G2/M phase from 15.9% on day 0 to 22.8% on day 2, and to 47.8% on day 3 in U373-MG cells. Similar results were observed in TMZ-sensitive U87-MG cells (data not shown). In TMZ-resistant T98G cells, the percentage of G2/M population was 13.8% on day 0, 19.3% on day 2, and 20.8% on day 3. These results indicate that TMZ induced G2/M arrest in malignant glioma cells, and that the extent of growth arrest depended on the sensitivity to TMZ.

### Downregulation of telomelase activity in TMZ-sensitive malignant glioma cells by TMZ

**Figure 3:**
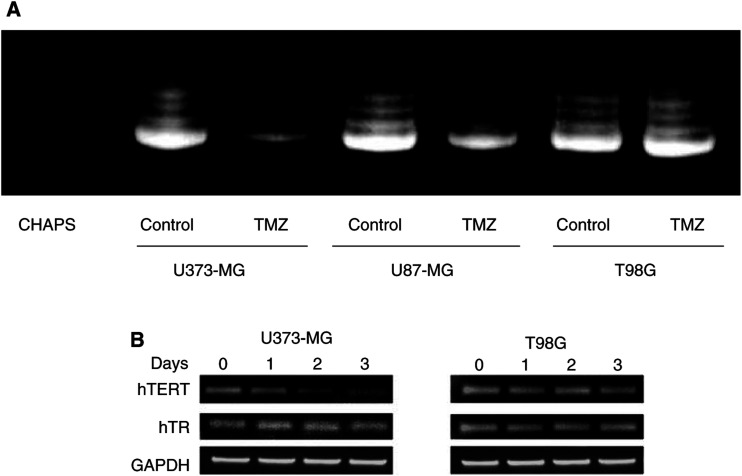
Downregulation of telomerase in TMZ-sensitive cells by TMZ. (**A**) Effect of TMZ on telomerase activity of U373-MG, U87-MG, and T98G cells. Telomerase activity was measured with the TRAPEZE™ Telomerase Detection Kit. Tumour cells were harvested 3 days after exposure to TMZ (100 *μ*M). Aliquots of 1 *μ*g protein extract were used for assay. The amplification products were applied to a 10% polyacrylamide gel and stained using SYBR Green. Lysis buffer CHAPS was used as a negative control. The data shown are representative of three independent experiments. (**B**) Effect of TMZ on expression of hTR or hTERT of U373-MG and T98G cells. RNA was isolated at the indicated time points from U373-MG or T98G cells treated with TMZ (100 *μ*M), RT–PCR was performed using primer sets for hTR or hTERT mRNA expression. The PCR products were run on a 2% agarose gel containing 0.5 *μ*g ml^−1^ ethidium bromide. Primer sets for GAPDH were used as an internal control. The data shown are representative of three independent experiments.

To investigate whether telomerase activity is affected by treatment with TMZ, we performed the telomerase activity assay (TRAP assay). As shown in Figure 3A

, telomerase activity of U373-MG and U87-MG cells was suppressed by treatment with TMZ (100 *μ*M) for 3 days. The intensity of telomerase activity in treated U373-MG and U87-MG cells was 10 and 15% of that in untreated tumour cells, respectively. In contrast, there was no significant decline in telomerase activity in T98G cells treated with TMZ (100 *μ*M). These results indicate that inhibition of telomerase activity correlated with the sensitivity of malignant glioma cells to TMZ.

### Inhibition of hTERT mRNA expression in TMZ-sensitive malignant glioma cells by TMZ

Since expression of hTERT mRNA is closely associated with telomerase activity (Meyerson *et al*, 1997; Nakamura *et al*, 1997), we examined whether hTERT mRNA expression is suppressed in U373-MG or U87-MG cells by TMZ. As shown in Figure 3B, the expression level of hTERT mRNA in U373-MG cells treated with TMZ (100 *μ*M) was decreased to 67% on day 1, 19% on day 2, and 25% on day 3 compared to the baseline value. A decline in hTERT mRNA was also detected in U87-MG cells treated with TMZ (data not shown). In contrast, the mRNA hTERT level of T98G cells following TMZ treatment remained unchanged. There was no decrease of hTR expression in U373-MG or T98G cells after TMZ treatment. These results indicate that inhibition of telomerase activity in TMZ-sensitive cells following TMZ treatment is due to a striking decrease in hTERT mRNA.

### Inhibition of hTERT transcription in TMZ-sensitive malignant glioma cells by TMZ

**Figure 4:**
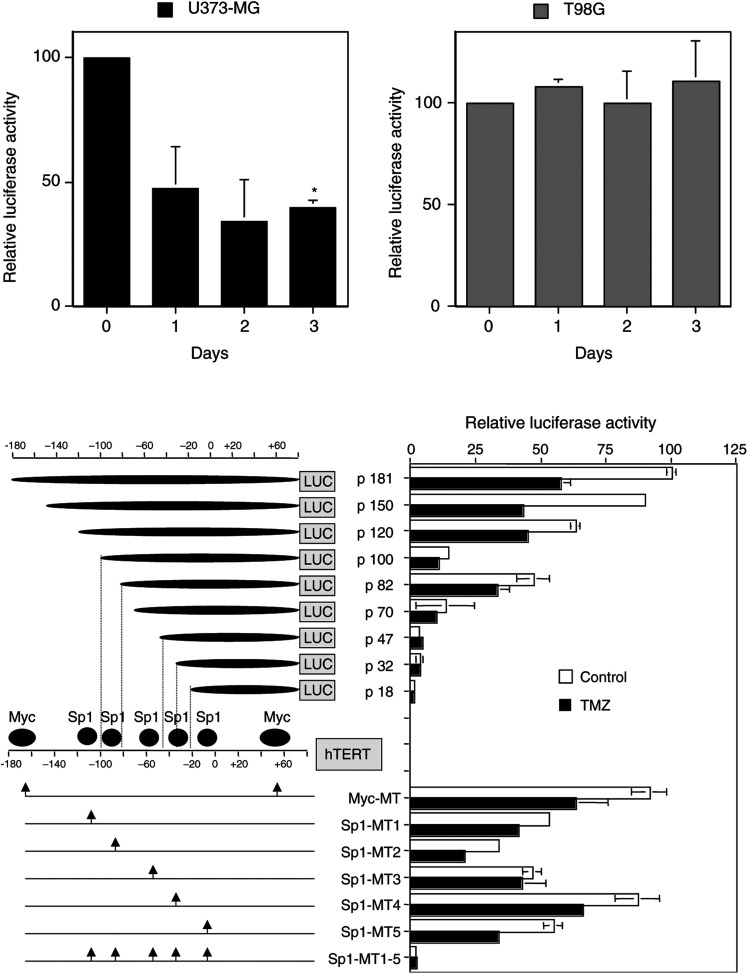
Inhibition of hTERT transcription in TMZ-sensitive cells by TMZ. (**A**) Effect of TMZ on transcriptional activity of hTERT core promoter (p181) in U373-MG and T98G cells. At 1 day after TMZ treatment (100 *μ*M), tumour cells were transfected with 0.5 *μ*g of luciferase expressing vector driven by hTERT core promoter using cationic lipid. At 48 h after the transfection, the cells were washed, lysed, and assayed for luciferase activity. The *β*-galactosidase reporter pCMV*β* was cotransfected as control for transfection efficiency. The relative luciferase activity of untreated U373-MG and T98G cells normalised by *β*-galactosidase activity was regarded as 100%. The results shown are the means±s.d. of three independent experiments. ^*^P=0.005. (**B**) Deletion and mutational analyses of the hTERT core promoter (p181). Luciferase reporter plasmids with serial deletions of the core promoter or substitution mutations in the factor binding sites were prepared. Arrows indicate the mutated sites for binding factors shown above the figure. These reporter plasmids were transfected into U373-MG cells 1 day after TMZ treatment (100 *μ*M), and luciferase assays were performed as described above. The *β*-galactosidase reporter pCMV*β* was cotransfected as control for transfection efficiency. The relative luciferase activity of the p181 core promoter in untreated U373-MG cells normalised by *β*-galactosidase activity was regarded as 100%. The results shown are the means±s.d. of three independent experiments.

Since TMZ significantly inhibited the expression of hTERT mRNA in U373-MG or U87-MG cells, we examined whether TMZ suppresses the transcriptional activity of hTERT. The proximal 181 bp region from a transcription start site is a core promoter of hTERT gene for transcriptional activation (Takakura *et al*, 1999). As shown in Figure 4A

, transcriptional activity of hTERT gene was decreased in U373-MG cells treated with TMZ (100 *μ*M) in a time-dependent manner. The luciferase activity of U373-MG cells using the p181 promoter was significantly suppressed to 45% (day 3) of that of untreated U373-MG cells (*P*=0.005). Similar results were observed in treated U87-MG cells (data not shown). In contrast, there was no significant reduction in transcriptional activity of T98G cells during TMZ treatment. These results indicate that TMZ inhibits hTERT at the transcriptional level and subsequently downregulates telomerase activity in TMZ-sensitive malignant glioma cells.

### TMZ inhibited the transcriptional activity of hTERT promoter via the Sp1 binding sites

The core promoter (p181) has two E-boxes that bind c-Myc and five GC-boxes for Sp1 binding (Figure 4B). When an E-box located at the 5′-end of the core promoter was deleted (p150), reduction in transcriptional activity was only marginal in untreated U373-MG cells. Abrogation of two E-boxes by mutation (Myc-MT) also led to only a slight reduction in transcriptional activity in untreated tumour cells. On the other hand, reduction in transcriptional activity of the p150 or Myc-MT promoter following treatment with TMZ was still significant compared to baseline (*P*<0.01). These suggest that E-boxes might not be essential for transcriptional activation in U373-MG cells and for TMZ-induced decline in telomerase activity. Further deletions of the core promoter p181 from −150 to −70 (p120, p100, p82, or p70) led to 60–92% reduction in transcriptional activity in untreated U373-MG cells. Additionally, the extent of TMZ-induced reduction in transcriptional activity of these promoters was less than that of p180 or p150 promoter. Moreover, the p47, p32, and p18 had no significant transcriptional activity in untreated U373-MG cells. These findings suggest that GC-boxes for Sp1 binding were more essential for transcriptional activity in U373-MG cells than E-boxes for c-Myc, and that TMZ might interfere with some Sp1 sites. Although abrogation of each Sp1 site by mutation except Sp1-MT4 reduced transcription to various extents, TMZ-induced reduction in Sp1-MT1, -MT2, -MT4, or -MT5 was distinct. There was no significant difference between control and TMZ treatment in Sp1-MT3. Mutation of all Sp1 sites (Sp1-MT1–5) led to a >95% loss of transcriptional activity in untreated U373-MG cells. These results indicate that Sp1 sites are more important for transcriptional activity in U373-MG cells than c-Myc sites and the reduction of transcriptional activity induced by TMZ might be due to TMZs interfering with Sp1 binding sites (at least Sp1 at −60).

### Effect of O^6^-BG on TMZ-resistant T98G cells

**Figure 5:**
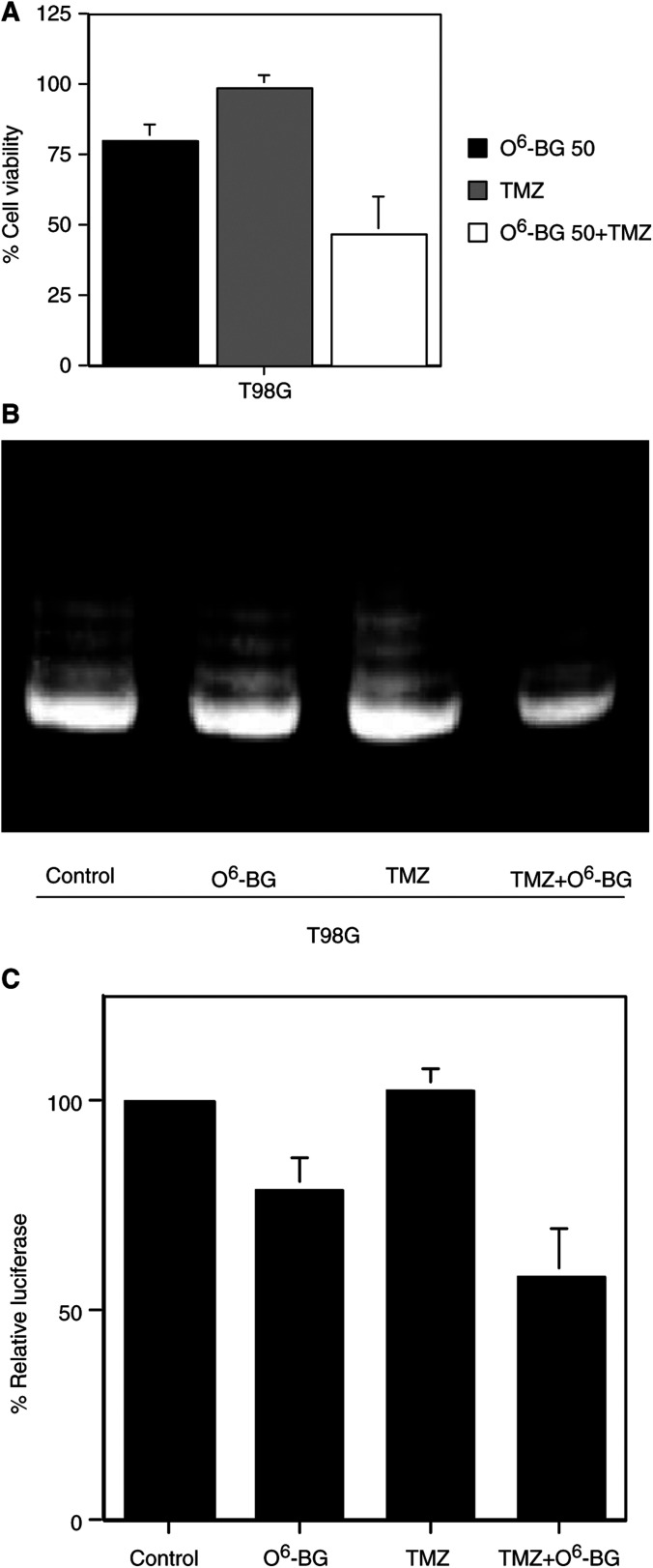
Effect of O^6^-BG on TMZ-resistant T98G cells. (**A**) Effect of combining TMZ with O^6^-BG on cell viability of T98G cells. Tumour cells were seeded at 2 × 10^3^ cells per well (0.1 ml) in 96-well flat-bottomed plates, and 24 h later treated with 0.1% DMSO or 50 *μ*M O^6^-BG for 2 h. At 3 days after exposure to TMZ (100 *μ*M), the cells were trypsinised and the number of viable cells was counted. The viability of the untreated cells was regarded as 100%. The results shown are the means±s.d. of three independent experiments. (**B**) Effect of combining TMZ with O^6^-BG on telomerase activity of T98G cells. Telomerase activity was measured with the TRAPEZE™ Telomerase Detection Kit. Tumour cells treated as described above were harvested 3 days after exposure to TMZ (100 *μ*M). Aliquots of 1 *μ*g protein extract were used for assay. The amplification products were applied to a 10% polyacrylamide gel and stained using SYBR Green. The data shown are representative of two independent experiments. (**C**) Effect of combining TMZ with O^6^-BG on transcriptional activity of hTERT core promoter (p181) in T98G cells. At 1 day after the treatment with TMZ (100 *μ*M) and/or O^6^-BG (50 *μ*M), tumour cells were transfected with 0.5 *μ*g of luciferase expressing vector driven by hTERT core promoter using cationic lipid. At 48 h after the transfection, the cells were washed, lysed, and assayed for luciferase activity. The *β*-galactosidase reporter pCMV*β* was cotransfected as control for transfection efficiency. The relative luciferase activity of untreated T98G cells normalised by *β*-galactosidase activity was regarded as 100%. The results shown are the means±s.d. of three independent experiments.

To investigate the effect of O^6^-BG on TMZ-resistant tumour cells, T98G cells were treated with TMZ in the presence of O^6^-BG. As shown in Figure 5A

, O^6^-BG significantly enhanced the antitumour effect of TMZ on T98G cells (*P*<0.01). Treatment with TMZ (100 *μ*M) and O^6^-BG (50 *μ*M) inhibited the viability of T98G cells to 47% of the control, although O^6^-BG alone showed some inhibitory effect. Then we investigated if telomerase activity is inhibited in T98G cells following treatment with TMZ and O^6^-BG. As shown in Figure 5B, combining TMZ with O^6^-BG inhibited telomerase activity in T98G cells. The intensity of telomerase activity in T98G cells treated with O^6^-BG alone or combining O^6^-BG with TMZ was 81 or 59% of that in untreated T98G cells. Moreover, treatment with O^6^-BG alone or combination of O^6^-BG with TMZ inhibited the transcriptional activity of hTERT to 78 or 57% of the control (Figure 5C). These results indicate that O^6^-BG not only sensitised T98G cells to TMZ, but also downregulated telomerase activity in T98G cells treated with TMZ.

## DISCUSSION

In the present study, we showed that treatment with TMZ inhibited telomerase activity in TMZ-sensitive tumour cells by interfering with binding sites of transcription factor Sp1. In contrast, telomerase activity remained unchanged in TMZ-resistant tumour cells during the treatment with TMZ. When TMZ-resistant tumour cells were sensitised to TMZ by O^6^-BG, a decline in telomerase activity was detected. These findings suggest that response of malignant glioma cells to TMZ can be monitored by reduction in telomerase activity. Recent investigation showed that radiation treatment decreases the telomerase activity in HeLa cells, which correlated with cell death (Sawant *et al*, 1999). Moreover, the decrease in telomerase activity following chemotherapy such as vincristine or doxorubicin paralleled the decrease in cell viability in several tumour cells (Faraoni *et al*, 1997; Lin *et al*, 2001). Therefore, determination of telomerase activity during or after treatment with chemotherapy or radiation may be a novel measure for monitoring chemo- or radioresponse of tumour cells.

On the other hand, the association between telomerase activity in untreated tumour cells and their chemosensitivity is controversial. Telomerase inhibition rendered breast cancer cells more susceptible to etoposide or doxorubicin, but not to cisplatin (Ludwig *et al*, 2001). Malignant glioma cells were sensitised to cisplatin by inhibition of telomerase (Kondo *et al*, 1998b). Furthermore, inhibition of telomerase resulted in increased sensitivity to cisplatin, but increased resistance to TMZ in melanoma cells (Tentori *et al*, 2003). Therefore, the relationship between levels of telomerase activity in tumour cells prior to anticancer therapies and their sensitivity to therapeutic agents may depend on the type of cell or treatment. Indeed, in the present study, TMZ-sensitive and -resistant malignant glioma cells express a similar high level of telomerase. As suggested by Tentori *et al* (2003), TMZ sensitivity seems to be dependent mainly on AGT activity rather than on telomerase levels in untreated tumour cells.

Regulation of telomerase activity is tightly controlled as it is expressed in the vast majority of malignant cells and in limited types of somatic cells (Kim *et al*, 1994; Chadeneau *et al*, 1995; Shay and Wright, 1996; Hiraga *et al*, 1998). Therefore, understanding the molecular mechanisms of telomerase activation or repression is an important step in the development of diagnostic or therapeutic applications. Telomerase activity is mainly regulated by hTERT gene expression (Meyerson *et al*, 1997; Nakamura *et al*, 1997). The hTERT promoter region has a high density of CpG dinucleotides and a number of putative transcription factor binding sites such as Sp1 or c-Myc (Horikawa *et al*, 1999; Takakura *et al*, 1999). Transcriptional activity of hTERT is dependent on the proximal 181 bp region of the promoter, which is essential for transactivation in immortalised and cancer cells (Takakura *et al*, 1999). This core promoter (p181) contains two E-boxes and five GC-boxes, the consensus binding sequences for c-Myc and Sp1, respectively. In the present study, our findings with serial deletion or mutation assays of the p181 promoter indicated that c-Myc sites within p181 were less important for transcriptional regulation of the hTERT gene in malignant glioma cells than Sp1 sites. A decline in telomerase activity by TMZ might result from a diminished level or function of Sp1. Our findings were consistent with recent investigation demonstrating that Sp1 sites are important for human papillomavirus E2-mediated repression of the hTERT promoter (Lee *et al*, 2002). On the other hand, it has been recently demonstrated that arsenic downregulates the promoter activity of hTERT by interfering with both c-Myc and Sp1 binding sites (Chou *et al*, 2001). Although Sp1 and c-Myc cooperatively activate hTERT expression (Kyo *et al*, 2000), it is possible that TMZ or E2 directly or indirectly suppresses the transactivation function of Sp1 itself.

Sp1 is one of the very first mammalian transcription factors to be cloned (Kadonaga *et al*, 1987). It binds to GC-rich sequences such as GC-boxes (Gidoni *et al*, 1985) and basic transcription elements (Imataka *et al*, 1992). Additionally, Sp1 binding sites are found in the promoters of many housekeeping genes (Black and Azizkhan-Clifford, 1999). Therefore, it has been widely accepted that Sp1 functions as a basal transcription factor and Sp1 binding sites represent constitutive promoter elements to support basal transcription in these promoters (Black *et al*, 2001). Recent investigations indicate that Sp1 sites are involved in growth regulation (Chen *et al*, 2000). In case of malignant glioma cells, inhibition of Sp1 sites-dependent transcription reduced cell growth as well as invasiveness (Ishibashi *et al*, 2000). Interestingly, there is increasing evidence demonstrating the interaction of Sp1 with GC-rich sequences within the p27^KIP1^ promoter (Grinstein *et al*, 2000). Inhibition of endogenous Sp1 activity by a dominant-negative Sp1 mutant was associated with cell cycle arrest and increased level of p27^KIP1^ (Grinstein *et al*, 2000). Since the cell cycle arrest (Figure 2) and the increased expression of p27^KIP1^ (data not shown) were detected in TMZ-sensitive U373-MG cells following TMZ therapy, we suggest that TMZ treatment may induce Sp1 repressors in TMZ-sensitive cells, resulting in inhibition of telomerase activity and increase of p27^KIP1^ expression to show antitumour efficacy.

DNA-damaging chemotherapeutic agents such as TMZ alter DNA at the O^6^ position of guanine and mediate their cytotoxicity at this site (Gerson, 2002). O^6^-alkylguanine DNA adducts are repaired by AGT. Tumour expression of AGT activity varies and correlates with therapeutic response to chemotherapy. Certainly, TMZ-sensitive U373-MG or U87-MG cells express a low level of AGT activity (Baer *et al*, 1993), while a high level of AGT is detected in TMZ-resistant T98G cells (Pieper *et al*, 1996). Although the AGT promoter contains Sp1 binding sites like the hTERT gene (Pieper *et al*, 1996; Horikawa *et al*, 1999; Takakura *et al*, 1999), the relationship between telomerase and AGT activity is unclear. Several possibilities may be raised. If TMZ produces enough Sp1 repressors to suppress AGT activity in T98G cells, the tumour cells would be sensitised to TMZ. If TMZ specifically suppresses Sp1 binding sites at the hTERT gene, the Sp1 activity of AGT gene would remain unchanged. It is also possible that it is a result of methylation at the G–C sequences either at guanine O^6^ or cytosine C^5^, which may inhibit Sp1 binding and downregulate the hTERT gene. Further investigations will be necessary to elucidate the precise mechanisms.

In summary, we demonstrate that a decline in telomerase activity by TMZ correlates with cellular sensitivity to TMZ. In TMZ-sensitive malignant glioma cells, telomerase activity was inhibited by treatment with TMZ. Telomerase activity of TMZ-resistant tumour cells was suppressed by combining TMZ with O^6^-BG. Our findings suggest that the response of malignant gliomas to TMZ therapy could be followed by determination of telomerase levels.
